# The mitochondrial-derived peptide humanin activates the ERK1/2, AKT, and STAT3 signaling pathways and has age-dependent signaling differences in the hippocampus

**DOI:** 10.18632/oncotarget.10380

**Published:** 2016-07-01

**Authors:** Su-Jeong Kim, Noel Guerrero, Gabriella Wassef, Jialin Xiao, Hemal H. Mehta, Pinchas Cohen, Kelvin Yen

**Affiliations:** ^1^ Leonard Davis School of Gerontology, University of Southern California, Los Angeles, CA, USA

**Keywords:** humanin, ERK1/2, AKT, STAT3, signaling pathway, Gerotarget

## Abstract

Humanin is a small secreted peptide that is encoded in the mitochondrial genome. Humanin and its analogues have a protective role in multiple age-related diseases including type 2 diabetes and Alzheimer's disease, through cytoprotective and neuroprotective effects both *in vitro* and *in vivo.* However, the humanin-mediated signaling pathways are not well understood. In this paper, we demonstrate that humanin acts through the GP130/IL6ST receptor complex to activate AKT, ERK1/2, and STAT3 signaling pathways. Humanin treatment increases phosphorylation in AKT, ERK 1/2, and STAT3 where PI3K, MEK, and JAK are involved in the activation of those three signaling pathways, respectively. Furthermore, old mice, but not young mice, injected with humanin showed an increase in phosphorylation in AKT and ERK1/2 in the hippocampus. These findings uncover a key signaling pathway of humanin that is important for humanin's function and also demonstrates an age-specific *in vivo* effect in a region of the brain that is critical for memory formation in an age-dependent manner.

## INTRODUCTION

Humanin is a 24 amino acid peptide that is encoded in the 16S rRNA gene within the mitochondrial genome and is the first member of a new class of mitochondrial derived peptides that includes MOTS-c and the SHLP peptides [1–3]. Humanin was first discovered as a neuroprotective molecule that protected neurons against amyloid-beta (Aβ) toxicity [4]. Our group and the Reed group then demonstrated that humanin has both an anti-apoptotic and cytoprotective function *via* interaction with IGFBP3 and Bax, respectively [5, 6]. Humanin is secreted from cells and thus humanin is detected in both conditioned medium and plasma [7, 8]. The plasma levels of humanin decline with age in mice and humans [9] and their levels are positively correlated with longevity in mouse models [10]. The long-lived Ames dwarf mice have a 50% increase in circulating humanin levels, whereas the short-lived GH-transgenic mice have lower levels [11–13]. Humanin and its analogues play a protective role in multiple age-related diseases including type 2 diabetes, cardiovascular disease, and stroke [8, 9, 14–16]. *In vivo* studies regarding humanin's neuroprotective role in Alzheimer's Disease (AD) mouse models showed that humanin administration to the triple transgenic mice, which contains three mutations associated with familial Alzheimer's disease, improved spatial learning while reducing memory deficits, Aβ plaque accumulation, and neuro-inflammatory response [17]. Because humanin is a secretory peptide, humanin participates in a number of diverse extracellular signaling pathways in addition to its intracellular regulatory function.

In terms of signaling, *in vitro* humanin treatment increases AKT-1 phosphorylation in mouse primary cortical neurons, and *in vivo* humanin injection also elevates AKT-1 phosphorylation after cerebral I/R injury while decreasing infarct volume [18]. In mouse heart, humanin injection increases AMPK phosphorylation [19]. Ying *et al*. showed that humanin binds to the formyl-peptide receptor-like receptor 1 (FPRL-1) to inhibit A-β induced cell death [20]. Upon activation by humanin, the receptor induces an increase of Ca^2+^ flux and activation of extracellular signal-regulated kinase (ERK 1/2) [20]. However, Hashimoto *et al.* showed that knockdown of the mouse counterpart of FPRL-1, FPR2, did not attenuate humanin's neuroprotective effect against AD-related insults, suggesting that there was another receptor for humanin other than FPR2 [21]. Their group demonstrated that the IL-6 receptor family subunits including the receptor for ciliary neurotrophic factor α (CNTFR-α), WSX-1, and glycoprotein 130kDa (GP130/IL6ST) mediate the neuroprotective role of humanin [22].

GP130 is a transmembrane protein and serves as the signal transduction unit of the IL-6 receptor family [23]. IL-6 binds to the α-receptor which does not itself signal, instead, it recruits two β-receptors and causes them to form a dimer. All IL-6 family cytokines use GP130 as a β-receptor. Dimerization of GP130 receptors induces the activation of janus kinases (JAK1 and JAK2), then subsequently activates signal transducer and activator of transcription 3 (STAT3) and STAT1 [24]. The dimerized STATs translocate to the nucleus and regulate transcription. The second signaling pathway mediated by GP130 recruits SHP-2. SHP-2 is phosphorylated by JAK and interacts with growth-factor receptor bound protein 2 (Grb2), which induces the activation of mitogen-activated protein kinase (MAPK) [24]. Additionally, GP130 activates the Src-family kinases and the PI3K/AKT signaling pathway [25, 26].

Extracellular signal-regulated kinase (ERK1/2), a member of the mitogen-activated protein kinase pathway, is involved in many fundamental cellular processes including cell proliferation, survival, differentiation, mobility, and apoptosis [27, 28]. An emerging role of ERK 1/2 suggests that it is involved in the pathophysiology of synaptic plasticity and memory formation *via* CRE-mediated transcription in the hippocampus [29, 30]. Another signaling molecule implicated in synaptic plasticity and memory formation is phosphoinositide 3-kinase (PI3K). PI3K is involved in AMPA (α-amino-3-hydroxy-5-methyl-4-isoxazolepropionic acid) receptor insertion to the postsynaptic membrane, activation of the ERK pathway, and initiation of protein synthesis [31].

Humanin protects against cellular stress and improves pathologies in multiple age-related diseases including AD and diabetes, and we have previously shown that humanin activates intracellular signaling in pancreatic beta cells [32]. Nevertheless, the signaling pathways underlying humanin's cytoprotective roles have yet to be elucidated in detail. Here, we characterize the humanin signaling pathway *in vitro* and *in vivo* in multiple models.

## RESULTS

### Ingenuity pathway analysis^TM^ (IPA) reveals a putative humanin mediated signaling pathway

**Figure 1:**
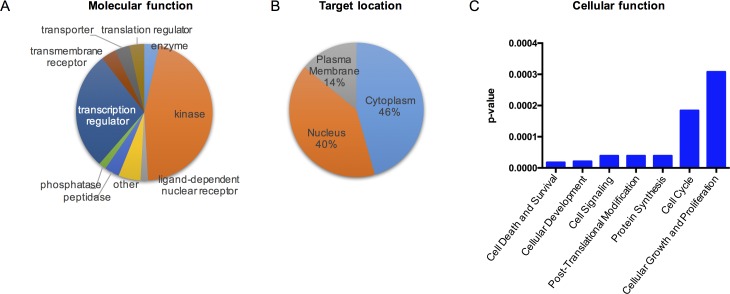
Deciphering the acute humanin signaling pathway Targets were identified if there was a significant minimum of 20% increase or decrease in phosphorylation. A pie chart showing humanin targets' (**A**) biological functions and (**B**) subcellular localization. The cytoplasm includes mitochondria, lysosome, and other subcellular compartment in the cytoplasm (**C**) Top molecular and cellular functional classes to which the humanin-targeted proteins are associated.

To determine the effect of humanin in signaling responses, we initially profiled the phosphorylated proteins in SH-SY5Y cells, a human neuroblastoma cell line, following 100μM HNG (a potent humanin analogue) treatment for 30min in serum free conditions by using the Phospho Explorer Antibody Array. HNG is generated by replacement of Ser14 with glycine, and this substitution increases humanin activity [4]. We found that HNG led to the significant phosphorylation of 57 proteins (Table 1). We then used Ingenuity^®^ Pathway Analysis (IPA) to attempt to identify the humanin signaling pathway. The HNG targets can be categorized into several different molecular functional types: kinase, transcription regulator, transmembrane receptor, and so on (Figure 1A). The HNG targets are broadly distributed in different subcellular compartments of the cell (Figure 1B). The IPA results suggested that humanin may exert a cytoprotective role through multiple cellular pathways including IGF-I, EGF, and PI3K/AKT pathways (Figures 1C, S1A, and S1B).

**Table 1:** The list of the proteins changed in Phospho Explorer Antibody Array

ID (phosphorylation)	Swiss Prot ID	p-value (HNG vs. Water)	Mean Ratio (HNG vs. Water)	Fold Change (HNG vs. Water)	Target Location	Molecular Function
MEK1 (Phospho-Ser298)	Q02750	0.00	1.47	1.47	Cytoplasm	kinase
GSK3 beta (Phospho-Ser9)	P49841	0.00	2.33	2.33	Nucleus	kinase
Shc (Phospho-Tyr349)	P29353	0.00	1.48	1.48	Cytoplasm	kinase
NFkB-p65 (Phospho-Ser276)	Q04206	0.00	0.74	−1.36	Nucleus	transcription regulator
VEGFR2 (Phospho-Tyr1214)	P35968	0.00	1.22	1.22	Plasma Membrane	kinase
HDAC8 (Phospho-Ser39)	Q9BY41	0.01	0.80	−1.25	Nucleus	transcription regulator
Tau (Phospho-Ser356)	P10636	0.01	1.62	1.62	Plasma Membrane	other
CREB (Phospho-Ser121)	P16220	0.01	0.66	−1.52	Nucleus	transcription regulator
IL-2RA/CD25 (Phospho-Ser268)	P01589	0.01	1.24	1.24	Plasma Membrane	transmembrane receptor
ATPase (Phospho-Ser16)	P05023	0.01	1.47	1.47	Plasma Membrane	transporter
Raf1 (Phospho-Tyr341)	P04049	0.01	0.73	−1.38	Cytoplasm	kinase
EGFR (Phospho-Tyr1016)	P00533	0.01	0.70	−1.42	Plasma Membrane	kinase
BCL-XL (Phospho-Thr47)	Q07817	0.01	2.31	2.31	Cytoplasm	other
PLC-beta (Phospho-Ser1105)	Q01970	0.01	1.39	1.39	Cytoplasm	enzyme
Interferon-gamma receptor alpha chain precursor (Phospho-Tyr457)	P15260	0.01	1.34	1.34	Plasma Membrane	transmembrane receptor
SRF (Phospho-Ser99)	P11831	0.02	1.27	1.27	Nucleus	transcription regulator
Caspase 2 (Phospho-Ser157)	P42575	0.02	0.68	−1.46	Cytoplasm	peptidase
p53 (Phospho-Ser46)	P04637	0.02	1.37	1.37	Nucleus	transcription regulator
Histone H2A.X (Phospho-Ser139)	P16104	0.02	1.53	1.53	Nucleus	transcription regulator
AMPK1 (Phospho-Thr174)	Q13131	0.02	1.84	1.84	Cytoplasm	kinase
Mnk1 (Phospho-Thr385)	Q9BUB5	0.02	1.57	1.57	Cytoplasm	kinase
EGFR (Phospho-Ser1070)	P00533	0.02	1.88	1.88	Plasma Membrane	kinase
IRS-1 (Phospho-Ser639)	P35568	0.02	1.82	1.82	Cytoplasm	enzyme
p53 (Phospho-Ser315)	P04637	0.02	1.97	1.97	Nucleus	transcription regulator
IkB-epsilon (Phospho-Ser22)	O00221	0.02	1.50	1.50	Nucleus	transcription regulator
Tau (Phospho-Ser235)	P10636	0.02	1.57	1.57	Plasma Membrane	other
ATF2 (Phospho-Thr71/53)	P15336	0.02	0.83	−1.21	Nucleus	transcription regulator
eIF4G (Phospho-Ser1108)	Q04637	0.02	1.30	1.30	Cytoplasm	translation regulator
Calmodulin (Phospho-Thr79/Ser81)	P62158	0.02	1.35	1.35	Cytoplasm	other
MSK1 (Phospho-Thr581)	O75582	0.02	1.23	1.23	Nucleus	kinase
STAT5B (Phospho-Ser731)	P51692	0.03	1.34	1.34	Nucleus	transcription regulator
FAK (Phospho-Ser910)	Q05397	0.03	1.79	1.79	Cytoplasm	kinase
Catenin beta (CTNNB) (Phospho-Tyr489)	P35222	0.03	0.66	−1.52	Nucleus	transcription regulator
GSK3 alpha (Phospho-Ser21)	P49840	0.03	2.96	2.96	Nucleus	kinase
MKK3 (Phospho-Ser189)	P46734	0.03	1.29	1.29	Cytoplasm	kinase
CDC2 (Phospho-Tyr15)	P06493	0.03	1.28	1.28	Nucleus	kinase
LYN (Phospho-Tyr507)	P07948	0.03	1.97	1.97	Cytoplasm	kinase
Cyclin D1 (Phospho-Thr286)	P24385	0.03	0.64	−1.56	Nucleus	transcription regulator
KSR (Phospho-Ser392)	Q8IVT5	0.03	1.43	1.43	Cytoplasm	kinase
PDGF R alpha (Phospho-Tyr849)	P16234	0.03	0.73	−1.37	Plasma Membrane	kinase
p21Cip1 (Phospho-Thr145)	P38936	0.03	0.69	−1.45	Nucleus	kinase
NFAT4 (Phospho-Ser165)	Q12968	0.03	0.66	−1.51	Nucleus	transcription regulator
PKC zeta (Phospho-Thr410)	Q05513	0.04	1.75	1.75	Cytoplasm	kinase
4E-BP1 (Phospho-Thr70)	Q13541	0.04	1.23	1.23	Cytoplasm	translation regulator
CDC25A (Phospho-Ser75)	P30304	0.04	1.54	1.54	Nucleus	phosphatase
p44/42 MAP Kinase (Phospho-Tyr204)	P27361/P28482	0.04	1.79	1.79	Cytoplasm	kinase
SEK1/MKK4 (Phospho-Thr261)	P45985	0.04	1.25	1.25	Cytoplasm	kinase
Synapsin (Phospho-Ser9)	P17600	0.04	1.30	1.30	Plasma Membrane	transporter
c-Jun (Phospho-Ser73)	P05412	0.04	0.64	−1.55	Nucleus	transcription regulator
STAT3 (Phospho-Ser727)	P40763	0.04	1.23	1.23	Nucleus	transcription regulator
Caspase 9 (Phospho-Ser196)	P55211	0.04	1.59	1.59	Cytoplasm	peptidase
SEK1/MKK4 (Phospho-Ser80)	P45985	0.04	1.25	1.25	Cytoplasm	kinase
STAT6 (Phospho-Thr645)	P42226	0.04	1.47	1.47	Nucleus	transcription regulator
Pyk2 (Phospho-Tyr580)	Q14289	0.04	1.32	1.32	Cytoplasm	kinase
CK2-b (Phospho-Ser209)	P67870	0.05	1.26	1.26	Cytoplasm	kinase
CDK5 (Phospho-Tyr15)	Q00535	0.05	2.30	2.30	Nucleus	kinase
Paxillin (Phospho-Tyr118)	P49023	0.05	0.77	−1.30	Cytoplasm	other
PAK1 (Phospho-Ser204)	Q13153	0.05	1.52	1.52	Cytoplasm	kinase
IkB-alpha (Phospho-Ser32/36)	P25963	0.05	1.23	1.23	Cytoplasm	transcription regulator
Estrogen Receptor-α (Phospho-Ser118)	P03372	0.05	1.27	1.27	Nucleus	ligand-dependent nuclear receptor

### Humanin rapidly induces the phosphorylation of AKT, ERK 1/2, and STAT3 in SH-SY5Y cells

**Figure 2:**
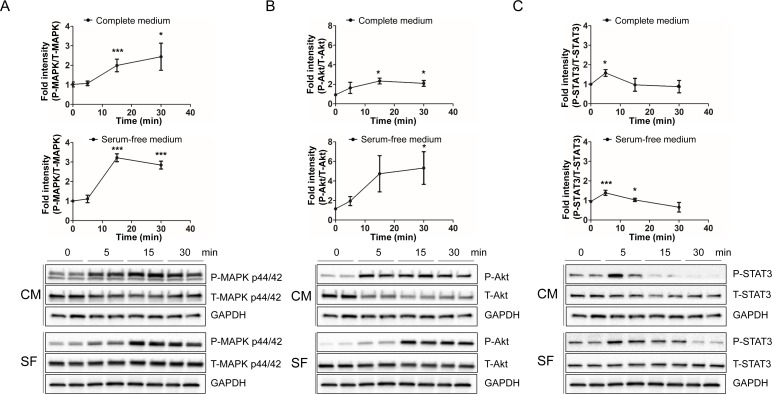
HNG rapidly increases the phosphorylation of MAPK p44/42 (ERK 1/2), AKT, and STAT3 in SH-SY5Y cells Quantification and representative western blots of (**A**) ERK activation, (**B**) AKT activation, and (**C**) STAT3 activation. Total cell lysates from SH-SY5Y cells following 100μM HNG treatment in DMEM or DMEM supplemented with 10% FBS for the indicated time periods were immunoblotted using anti-phospho- and total-ERK 1/2 (Thr^202^/Tyr^204^), AKT (Ser^473^), and STAT-3 (Tyr^705^). A densitometric analysis of ERK, AKT, and STAT-3 levels were performed using image J. Data are reported as mean ± SEM of three to six independent experiments. ***p < 0.001, **p < 0.01, *p < 0.05. Abbreviations: CM, Complete medium; SF, Serum-free medium.

IPA canonical pathway analysis suggested that the IL-6, JAK/STAT3, and PI3K/AKT pathways were activated in response to treatment with HNG. ERK, AKT, and STAT3 were activated as a common component in those signaling pathways. To validate the predicted humanin signaling pathway in SH-SY5Y cells, we treated them with 100μM HNG in serum free or complete media for the indicated time periods. We then analyzed the phosphorylation of AKT at Ser^473^ and Thr^308^, MAPK p44/42 (ERK 1/2) at Thr^202^/Tyr^204^, and STAT3 at Tyr^705^ and Ser^727^. We examined MAPK p44/42 as it is a common downstream pathway of both IL-6 and PI3K/AKT as shown in the IPA canonical pathway analysis. HNG treatment caused a rapid increase in phosphorylation of ERK 1/2 at its regulatory Thr^202^/Tyr^204^ site in both serum free and complete media condition (Figure 2A). Because of the robust effect on signaling using 100μM of HNG, we tested whether ERK phosphorylation could be increased with a lower dose of HNG in SH-SY5Y and HEK293 cells. Even 1nM of HNG showed an increase in phosphorylation of ERK 1/2 (Figures S3A and S3B). HNG treatment also rapidly increased PI3K-AKT and mTORC2-AKT signaling, as shown by an increase in phosphorylation of AKT at its regulatory Thr^308^ and Ser^473^ site, respectively (Figures 2B, S2, and S5A). HNG signaling in serum free media conditions potently increased AKT phosphorylation levels compared to the complete media conditions possibly because the basal activation of AKT may be lower in serum free-media. HNG treatment showed a potent increase in phosphorylation of STAT3 within 5 minutes and a decrease after 15 minutes at its regulatory Tyr^705^ site (Figure 2C), whereas HNG treatment did not show an increase in phosphorylation of STAT3 at its regulatory Ser^727^ site (data not shown). Dimerization and DNA binding of STAT3 require phosphorylation of its Tyr^705^ site and mitochondrial localization of STAT3 requires phosphorylation of its Ser^727^ site [33, 34]. This suggests that HNG treatment causes the nuclear localization of STAT3, but not mitochondrial localization.

### Humanin rapidly induces the phosphorylation of ERK 1/2 in HEK293 cells

**Figure 3:**
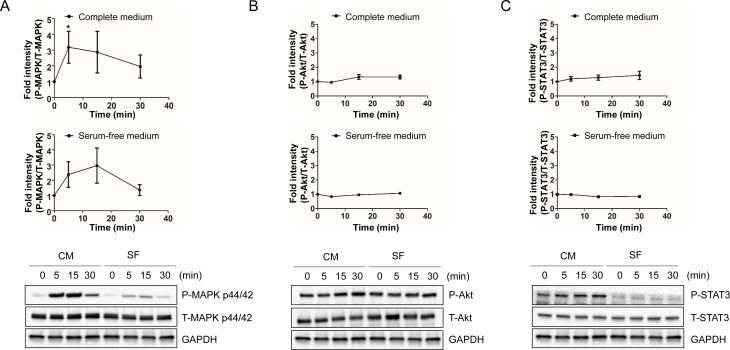
HNG rapidly increase the phosphorylation of MAPK p44/42 (ERK1/2) in HEK293 cells Quantification and representative western blots of (**A**) ERK activation, (**B**) AKT activation, and (**C**) STAT3 activation. Total cell lysates from HEK293 cells following 100μM HNG treatment for the indicated time periods were immunoblotted using anti-phospho- and total-ERK 1/2 (Thr^202^/Tyr^204^), AKT (Ser^473^), and STAT3 (Tyr^705^). A densitometric analysis of ERK, AKT, and STAT3 levels were performed using Image J. Data are reported as mean ± SEM of four independent experiments. *p < 0.05. Abbreviations: CM, Complete medium; SF, Serum-free medium.

We next looked at a different cell line to see if our observed effects were cell line specific. Therefore, we examined both the PI3K/AKT and JAK/STAT3 pathways in HEK293 cells. HEK 293 cells were treated with 100μM HNG for the indicated time periods and were immunoblotted using anti-phospho- and total-ERK 1/2 (Thr^202^/Tyr^204^), AKT (Ser^473^), and STAT3 (Tyr^705^). HNG treatment showed a rapid increase in phosphorylation of ERK 1/2 at its regulatory Thr^202^/Tyr^204^ site in both serum free and complete media conditions (Figure 3A). Complete media conditions showed higher levels of phosphorylation compared to serum free media, and it suggests that additive factors from serum synergistically activate ERK with HNG in HEK293 cells. In contrast to SH-SY5Y cells, HNG treatment did not show an increase in phosphorylation of AKT and STAT3 in both serum free and complete media condition (Figure 3B and 3C). Thus, these results suggest that humanin may activate different receptors in SH-SY5Y and HEK293 cells.

### PI3K, MEK, and JAK are involved in humanin-mediated AKT, ERK, and STAT3 activation

**Figure 4:**
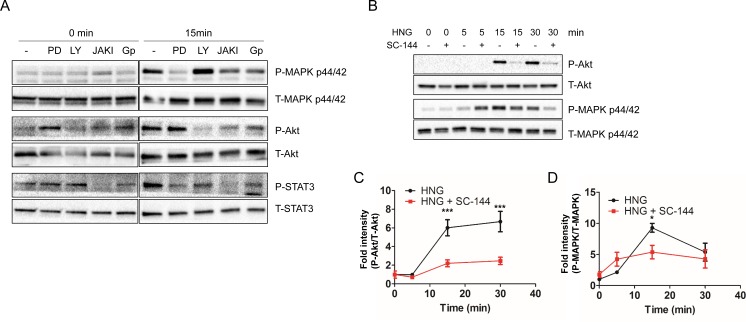
Inhibitors block the HNG-mediated activation of ERK, AKT, and STAT3. ( **A**) Representative western blots showing ERK, AKT, and STAT3 activation in conjunction with specific inhibitors under serum free conditions (*n* = 3). Quantification of (**C**) AKT and (**D**) ERK activation. Data are reported as mean ± SEM of 4 independent experiments. ***p < 0.001, *p < 0.05. Abbreviations: PD, PD98059; LY, LY294002; JAKI, JAK inhibitor I; GP, Anti-GP130 antibody.

As the humanin signaling pathway(s) has not yet been fully determined, we examined whether the canonical pathways regulate humanin-mediated AKT, ERK 1/2, and STAT3 activation. To accomplish this, we used specific inhibitors, LY294002, PD98059, and JAK I, to block PI3K, MEK, and JAK signaling, respectively. SH-SY5Y cells were pretreated with 10 μM LY294002, 10 μM PD98059, or 1μM JAK I for 30 min, followed by HNG treatment in serum free condition. LY294002 significantly inhibited phosphorylation of AKT, but not ERK (Figure 4A). PD98059 inhibited phosphorylation of ERK, but not AKT (Figure 4A). These results suggest that LY294002 and PD98059 specifically inhibited the activation of PI3K and MEK, and that PI3K and MEK are involved in HNG-induced AKT and ERK activation, respectively. We also determined whether HNG-mediated activation of STAT3 was dependent on AKT and ERK. LY294002 and PD98059 inhibited phosphorylation of STAT3, which indicated that humanin-induced activation of STAT3 was dependent on both PI3K and MEK (Figure 4A). Next, we examined the involvement of JAK, a well-defined upstream kinase of STAT3 [35]. JAK I inhibited STAT3 activation as well as AKT and ERK.

### GP130 is involved in humanin-mediated AKT, ERK, and STAT3 activation

JAK is constitutively associated with GP130, and humanin is known to bind to the trimeric receptor, which is comprised of IL-6 receptor family subunits including GP130, CNTFR alpha, and WSX-1 [22, 36]. In addition, IPA canonical pathway analysis showed that HNG activates IL-6 signaling. Thus, we examined whether GP130 is involved in humanin-induced AKT, ERK, and STAT3 activation. Humanin treatment in the presence of an anti-GP130 antibody inhibited phosphorylation of STAT3, AKT, and ERK (Figure 4A). We further confirmed the involvement of GP130 in the activation of AKT, ERK, and STAT3 by treating cells with SC-144, a GP130 chemical inhibitor. 20 μM SC-144 inhibited phosphorylation of AKT and ERK in SH-SY5Y cells (Figure 4B, 4C, and 4D). Compared to inhibition of AKT phosphorylation, a substantial amount of ERK phosphorylation is still detected in the presence of SC-144. Trifluoroacetic acid (TFA) is often used in the final cleavage of customized peptides from the solid support after solid phase synthesis. Thus, TFA is a common contaminant left behind in the final peptide preparation, even after purification. Trace amounts of TFA may affect the results of cell culture assays as has been previously reported [37]. We thus examined whether TFA increases ERK phosphorylation independent of the GP130 mediated humanin signaling pathway, and if the substantial amount of ERK phosphorylation in the presence of SC-144 is due to TFA-mediated ERK phosphorylation. 5μM TFA treatment showed a rapid increase in phosphorylation of ERK 1/2, but not AKT and STAT3 (Figure S4A). We then substituted hydrochloric acid for TFA during peptide synthesis and found that TFA-free humanin still increased the phosphorylation of ERK in both SH-SY5Y and HEK293 cells (Figures S4B and S4C). These results suggest that humanin-mediated ERK phosphorylation is independent of TFA. Although TFA-mediated ERK activation may lead to a substantial amount of ERK phosphorylation in the presence of SC-144, there is still the possibility that another receptor instead of GP130 may be involved in ERK phosphorylation in response to humanin (Figure 7). In fact, HEK293 cells have altered IL-6 signaling due to their lack of soluble IL-6R [38]. IPA pathway analysis suggested that a receptor tyrosine kinase (RTK) including EGFR and ErbBR may be involved in the humanin-mediated signaling pathway. Thus, we used the PathScan RTK Signaling Antibody Array kit to simultaneously detect phosphorylation of 28 receptor tyrosine kinases and 11 important signaling molecules. The array detected humanin-mediated AKT and ERK phosphorylation, but did not show an increase of any RTK in the array (Figures S5A and S5B). Further studies are required to reveal the alternative receptor that humanin binds and activates the signaling pathway involving ERK.

**Figure 5:**
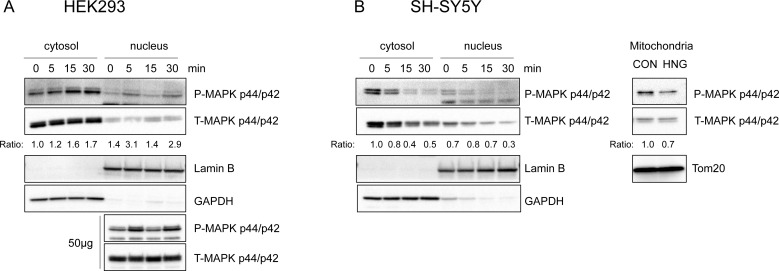
Phosphorylated ERK 1/2 is localized to cytosol, nucleus, and other subcellular compartments upon HNG treatment Representative western blots showing ERK1/2 subcellular localization (*n* = 3). (**A**) HEK293 cells were treated with 1μM HNG. Both cytosolic (15μg) and nuclear (15μg and 50μg) lysates were immunoblotted using phospho- and total-ERK antibody. (**B**) SH-SY5Y cells were treated with 100μM HNG. Mitochondria fractions were obtained 30min after HNG treatment. Cytosolic, nuclear, and mitochondrial (15μg) lysates were immunoblotted using phospho- and total-ERK antibody. Anti-GAPDH, Anti-Lamin B, anti-Tom20 antibody were used as the cytosolic, nuclear, mitochondrial fraction markers, respectively. The ratio is the fold change in phospho-MAPK normalized to total-MAPK compared to the cytosolic, time 0 phospho to total ratio.

### Phosphorylated ERK is localized to specific subcellular compartments

The subcellular localization of ERK plays a crucial role in its signaling function, particularly in achieving its specificity [39]. ERK is localized primarily in the cytoplasm. Upon stimulation, ERK is phosphorylated and becomes detached from its anchoring proteins to allow the translocation of ERK to other subcellular compartments. These include the nucleus, mitochondria, endosomes/lysosomes, various membranes, and cytoplasm, where it interacts with its substrates. To understand the target of humanin-mediated ERK activation, we examined the subcellular localization of ERK in the presence of humanin. In HEK293 cells, phosphorylated ERK increased in the cytoplasm and nucleus, but not in the mitochondria (Figure 5A). Interestingly, phosphorylated ERK decreased in the cytoplasm, nucleus, and mitochondria in SH-SY5Y cells (Figure 5B). These results suggest that phosphorylated ERK may play a different role in different cell types. Hence, further studies are required to understand the role of phosphorylated ERK in other subcellular compartments of SH-SY5Y cells.

**Figure 6:**
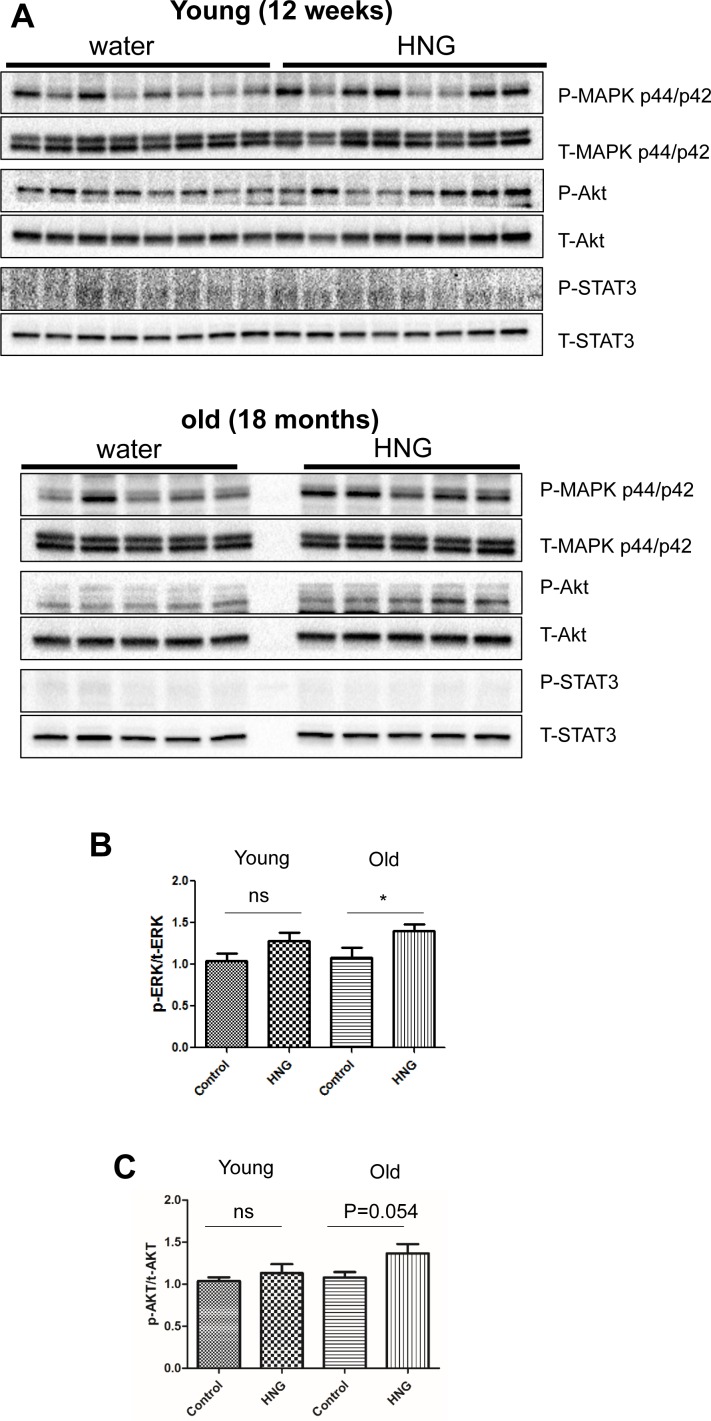
HNG increases AKT and ERK phosphorylation in the hippocampus of old mice (**A**) Western blot analysis for phospho- and total-AKT, ERK, and STAT3 in hippocampus of 12-week-old mice and 18-month-old mice. Quantitation of (**B**) normalized ERK activation and (**C**) normalized AKT activation in response to HNG. Total protein was used to normalize protein loading. Data are reported as mean ± SEM (*n* = 8 for 12-week-old mice, *n* = 5 for 18-month-old mice). *p < 0.05, ns, not significant.

### Humanin induces the phosphorylation of AKT and ERK 1/2 in the hippocampi of old mice

The ERK signaling pathway regulates a variety of cellular processes including proliferation, differentiation, and cellular metabolism. In the hippocampus, ERK activation is involved in synaptogenesis and memory function. Niikura *et al*. showed that humanin injection into 3xTg AD mice improves spatial learning and memory deficits, while reducing Aβ plaque accumulation and the neuro-inflammatory response [17]. To examine whether IP injection of HNG could affect signaling in the hippocampus of mice, which could support the enhanced cognitive function in AD mice, we injected humanin into young (12 weeks) and old (18 months) C57BL/6 male mice for 2 weeks and dissected the hippocampus and hypothalamus for immunoblotting. HNG treatment increased phosphorylation of AKT and ERK 1/2, but not STAT3 in the hippocampus of old mice only (Figure 6). HNG treatment did not show an increase in phosphorylation of AKT, ERK 1/2, or STAT3 in hypothalamus of either age group (Figure S6). These results demonstrate that humanin activates ERK and AKT signaling pathways, which could increase synaptic proteins and enhance memory function in old mice.

**Figure 7:**
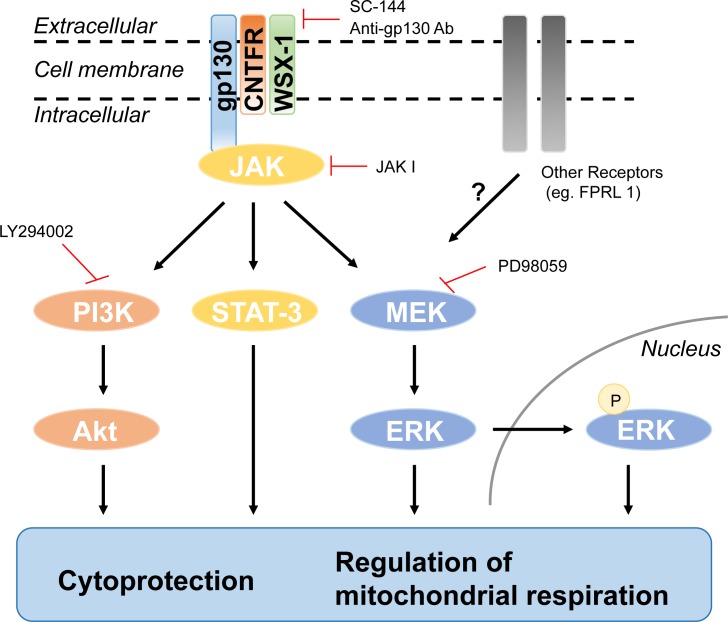
Schematic diagram of the humanin-mediated signaling pathway and its biological functions

## DISCUSSION

Humanin levels decline with age in tissue and plasma of both, rodents and humans [9]. Mitochondrial defects during aging and age-related diseases may explain this decrease in humanin level [40–42]. In fact, damage to the humanin encoding region at the mitochondria 16S rRNA was detected in age-related macular degeneration [43]. Humanin protects from several age-related diseases, but the underlying signaling pathways have not been thoroughly deciphered [44]. In this paper, we find that humanin activates the GP130 receptor and its canonical downstream signaling cascades of AKT, ERK 1/2, and STAT3. In cell types with altered GP130 signaling, we find that humanin can still activate ERK signaling, possibly by the FPRL receptor or internalization leading to intracellular signaling [20, 45]. Furthermore, *in vivo* studies, demonstrate that humanin can specifically stimulate AKT and ERK 1/2 in the hippocampi of older mice, restoring the levels back to a youthful state, counteracting the decline in AKT phosphorylation with age [46]. We find that humanin has differential signaling in different regions in the brain as well as in the liver (data not shown). This could be due to a number of reasons, such as differential expression of different GP130 co-receptors such as WSX-1 and CNTFR.

Notably, we find that the levels of phosphorylated AKT and ERK 1/2 increase in the hippocampi of older mice upon HNG treatment. These results suggest that humanin could improve cognitive function during aging and age-related diseases like Alzheimer's disease through these signaling pathways [46]. Why humanin only effects signaling in old mice is still unknown, but it is possible that humanin may have improved brain availability due to the leakier blood brain barrier found in old mice [47]. Alternatively, endogenous humanin levels are higher in young mice and these pathways may already be activated, while the lower endogenous humanin levels in old mice, associated with lower levels of activation, allow for a more notable induction (Figures S7A and S7B). Further studies to examine the increase of synaptic proteins, enhanced synaptic junctions, and cognitive function in mice co-injected with humanin and specific inhibitors for humanin-mediated signaling pathway will support the direct role of humanin-mediated signaling pathway in the hippocampus.

Our study implicates GP130 signaling as a key player in the beneficial effects of humanin action, and identifies humanin as a major GP130 agonist. In fact, transient activation of GP130 has many overlapping effects with humanin administration such as protection from doxorubicin toxicity, protection from ischemia/reperfusion injury, and metabolic effects including increasing insulin sensitivity [48–51]. For aging and age-related diseases the role of inflammation is well established with the term “inflammaging” being often used to describe the paradigm [52–54]. Although the beneficial effects of humanin administration counterintuitively activates GP130, which would increase inflammation and would therefore have a negative effect, this could highlight the difference between constitutive activation of inflammatory receptors *versus* transient activation. It is also possible that the different ligands that engage GP130 have different effects on cellular signaling. Alternatively, humanin's *in vivo* effects on IGF-I signaling could be the primary mediator of these beneficial effects. Further studies will be needed to examine this in more detail and dissect apart the possible therapeutic uses of humanin.

## MATERIALS AND METHODS

### Reagents and antibodies

HNG (a potent analogue of humanin with a glycine substitution, S14G) and humanin were synthesized and received from Genscript (Piscataway, NJ, USA) and New-England Peptide (Gardner, MA, USA). HNG was dissolved in Milli-Q water. PD98059, LY294002, JAK inhibitor I (EMD Millipore, Billerica MA, USA), and SC-144 hydrochloride (Sigma, St. Louis, MO, USA) were used for the inhibitor experiments. The following antibodies were used in this study: anti-AKT (pan) antibody (Cat. #4691S), anti-phospho-AKT (Ser473) antibody (Cat. #4060S), anti-phospho-AKT (Thr308) antibody (Cat. #13038P), anti-p44/42 MAPK (Erk1/2) antibody (Cat. #9102S), anti-phospho-p44/42 MAPK (Erk1/2)(Thr^202^/Tyr^204^) antibody (Cat. #4370S), anti-STAT3 antibody (Cat. #8204), anti-phospho-STAT3 (Tyr^705^) antibody (Cat. # 8204), anti-phospho-STAT3 (S^727^) antibody (Cat. #9134S), anti-GAPDH antibody (Cat. #5174S), and anti-Lamin B1 antibody (Cat. #12586). These antibodies are manufactured by Cell Signaling Technology (Danvers, MA, USA). Rabbit anti-GP130 antibody (Cat. #SC-655) and mouse anti-Tom20 antibody (Cat. #SC-17764) (Santa Cruz Biotechnology, Dallas, Texas, USA) were also used.

### Cell culture and treatment

HEK293 and SH-SY5Y cells were cultured in high glucose Dulbecco's modified Eagle's medium (DMEM; Life Technologies, Waltham, MA, USA) supplemented with 10% fetal bovine serum (FBS; Omega Scientific, Tarzana, CA, USA) at 37°C in 5% CO_2_. Cells were grown in complete media for 24 hrs. Prior to the peptide treatment in serum free condition, the cells were switched to DMEM for 2hrs followed by addition of the peptide for indicated time periods. For the inhibitor experiments, 10μM PD98059, 10μM LY294002, 1μM JAK inhibitor I, or 20μM SC-144 was added for 30 minutes followed by the peptide in DMEM or DMEM supplemented with 10% FBS incubated for the indicated time. The anti-GP130 antibody was co-treated with the peptide.

### Subcellular fractionation

After peptide treatment for the indicated time periods, cytosolic and nuclear fractions of HEK293 and SH-SY5Y cells were obtained as previously described with some modifications [55]. Briefly, cells pellets were harvested by centrifugation at 500 x g, washed in ice-cold PBS once and re-suspended in ice-cold fractionation buffer (10mM HEPES pH7.6, 3mM MgCl2, 10mM KCl, 5% (v/v) glycerol, 1% Triton X-100, and Halt protease & phosphatase inhibitor cocktail (ThermoFisher Scientific)) for 10min on ice. The supernatant (cytosolic fraction) was collected by centrifugation at 250 x g for 5min at 4°C. The cytoplasmic fraction was further centrifuged at 18,000 x g for 10min to obtain a cleared cytoplasmic fraction. The pellet was washed in ice-cold wash buffer (10mM HEPES pH 7.6, 1.5mM MgCl2, 10mM KCL, and Halt protease & phosphatase inhibitor cocktail) and centrifuged at 250 x g for 5min at 4°C. The pellet was then re-suspended in ice-cold nuclear extraction buffer (20mM HEPES pH 7.6, 1.5mM MgCl2, 420 mM NaCl, 25% (v/v) glycerol, 0.2mM EDTA, and Halt protease & phosphatase inhibitor cocktail) and sonicated for 30min in ice water, replenishing with crushed ice every 10min. The supernatant (nuclear fraction) was collected by centrifugation at 18,000 x g for 10min at 4°C. For the mitochondrial fraction, cells pellets were harvested by centrifugation at 500 x g for 5min, re-suspended in ice-cold mitochondria isolation buffer (70mM sucrose, 220mM D-Mannitol, 2mM HEPES pH 7.6, 0.5mg/ml defatted bovine serum albumin, buffer is adjusted to pH 7.4 with KOH just prior to use), and homogenized with fifteen passes in a prechilled glass-Teflon homogenizer. Nuclei and unbroken cells were discarded after centrifugation at 600 x g for 10min at 4°C. The mitochondria-rich fraction corresponded to the pellet obtained after centrifugation at 7,000 x g for 10min at 4°C.

### Western blot analysis

Cells were lysed with RIPA buffer (50mM Tris-HCl, pH7.4, 1% NP-40, 0.25% sodium deoxycholate, 150mM NaCl, 1mM EDTA) containing 0.1% SDS plus the Halt protease & phosphatase inhibitor cocktail. The lysates were homogenized using a sonicator, and the supernatant was collected by centrifugation at 15,000 x g for 15min at 4°C. For tissue, dissected hippocampi were lysed with RIPA buffer and homogenized using a tissue homogenizer followed by sonication. The supernatant was collected by centrifugation at 15,000 x g for 15min at 4°C. Protein content in the cellular lysates was quantified using the Pierce™ BCA Protein Assay Kit (ThermoFisher Scientific). Predetermined amounts of proteins (10-30μg) were separated on 4-20% SDS-PAGE gels and blotted onto PVDF membranes (Biorad, Hercules, CA, USA). Membranes were incubated with primary antibody at 4°C overnight according to the manufacturer's instructions. After several washes with Tris-buffered saline containing 0.1% Tween-20, membranes were incubated at room temperature (RT) for 1hr with the appropriate HRP-conjugated secondary antibody. Enhanced chemiluminescence was used for detecting specific bands. Membranes were imaged on a Bio-Rad ChemiDoc XRS^+^ imager. If necessary, relative intensities of band in each condition were measured using Image J, a free software program provided by National Institute of Health (Bethesda, Maryland, USA).

### PathScan RTK signaling antibody array

A PathScan RTK Signaling Antibody Array kit (Cell signaling Technology) was used according to the manufacturer's instructions. Briefly, cells were washed with ice-cold PBS and lysed in 1X Cell Lysis Buffer to collect cell lysates of control and HNG treated sample for the indicated time. The supernatant was collected by centrifugation at 15,000 x g for 15min at 4°C. Protein content in the cellular lysates was quantified using a Pierce™ BCA Protein Assay Kit. The Array Blocking Buffer was added to the array wells for 15min and predetermined amounts of protein (50μg) were incubated at 4°C overnight. After washing the array wells, the Detection Antibody Cocktail was added to the wells and incubated for 1h at RT followed by incubation with horseradish peroxidase-linked streptavidin for 30min at RT. The plates were then covered with the LumiGLO/peroxide reagent and imaged in the Bio-Rad ChemiDoc XRS^+^ imager.

### Animals

Male C57BL/6 mice at 12 weeks (*n* = 16) and 18 months (*n* = 10) of age were obtained from Jackson Laboratory (Bar Harbor, ME USA) and the National Institute of Aging (NIA), respectively. The mice were singly housed under standard 12-hr light-dark cycle with access to water and rodent food ad libitum (LabDiet, MO). Mice were randomly assigned to one of two experimental groups: a control group receiving daily Intraperitoneal (IP) injection of vehicle (sterilized water); a HNG-treated group receiving daily IP injection of 5mg HNG per kg body weight. Mice were euthanized after two weeks of treatment. Prior to euthanasia, these mice were fasted for 9 hours and were injected with HNG 15-30 minutes just before anesthesia with isoflurane. Tissues were collected, flash-frozen in dry ice and stored at −80°C.

### Pathway analysis of humanin-mediated signaling pathway

The specific humanin-mediated signaling pathways identified using the Phospho Explorer Antibody Array (Full moon Biosystems, Sunnyvale, CA, USA) were analyzed using Ingenuity^®^ Pathway Analysis (IPA; QIAGEN, Redwood city, CA, USA). A spreadsheet containing the list of humanin-mediated phosphorylated proteins was generated by using Partek^®^ Genomomics Suite (Partek Incorporated, St. Louis, Missouri, USA) and was uploaded into IPA. The criteria for the selection of the proteins of interest were an absolute fold change > 1.2 between control and HNG treated groups and *P* < 0.05. The software mapped each of the proteins to the repository of information in the Ingenuity Pathways Knowledge base. Molecular networks and canonical pathways regulated by humanin were obtained using IPA core analysis. Category rankings were based on the p values derived from the Fisher's exact test and the cut-off threshold for significance was a *P*-value < .05. Ratios were calculated as follows: number of genes in a given pathway that meet the cutoff criteria, divided by the total number of genes that make up that pathway and that are in the reference gene set.

### Statistical analysis

Data are presented as mean ± S.E.M. Significant differences were determined by Student's *t*-tests, one-way ANOVA followed by Tukey's *post hoc* test, and two-way ANOVA followed by Bonferroni post-tests using GraphPad Prism 5 software. Values of * < 0.05, ** < 0.01, *** < 0.001 were considered statistically significant.

## SUPPLEMENTARY MATERIAL


