# Supplementary material for: The mitochondrial-derived peptide humanin activates the ERK1/2, AKT, and STAT3 signaling pathways and has age-dependent signaling differences in the hippocampus
Source: Oncotarget. 2016 Jul 1;7(30):46899–912. doi: 10.18632/oncotarget.10380 (PMC5216912; doi:10.18632/oncotarget.10380)
Supplement: Supplementary file 1 [file oncotarget-07-46899-s001.pdf]

The mitochondrial-derived peptide humanin activates the ERK1/2, AKT, and STAT3 signaling pathways and has age-dependent signaling differences in the hippocampus

Supplementary Material

A

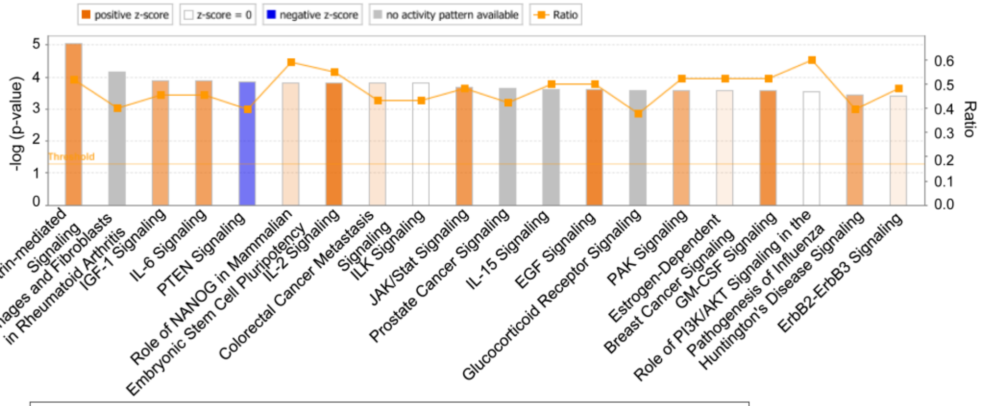

B

List of Genes associated with IGF-1 signaling
caspase 9
casein kinase 2, beta polypeptide
insulin receptor substrate 1
jun proto-oncogene
mitogen-activated protein kinase kinase 1
mitogen-activated protein kinase 1
mitogen-activated protein kinase 3
protein kinase C, zeta
protein tyrosine kinase 2
paxillin
Raf-1 proto-oncogene, serine/threonine kinase
SHC (Src homology 2 domain containing) transforming protein 1
serum response factor
signal transducer and activator of transcription 3 (acute-phase response factor)

Supplemental Figure 1: Top canonical pathways to which the humanin-targeted proteins are associated. (A) The high-raking categories are displayed along the x axis in decreasing order of significance. The y axis displays the – (log) p values. The orange points connected by a line represent the ratio. Based on the z-score, the orange indicates activation, blue inactivation, white have a z-score at or very closed to 0, and gray bars indicate pathways where no prediction can currently be made. (B) A list of genes associated with IGF-1 signaling.

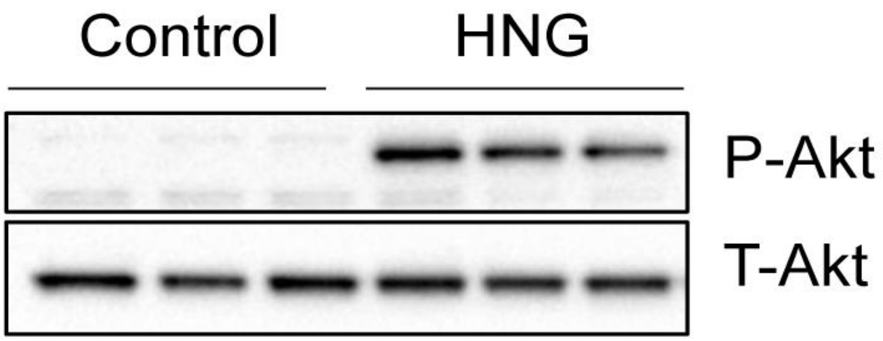

Supplemental Figure 2: HNG increases AKT (Thr^{308}) phosphorylation in SH-SY5Y cells. Western blot analysis for phospho- AKT at Thr^{308} and total-AKT. Cells were treated with 100µM HNG for 30min.

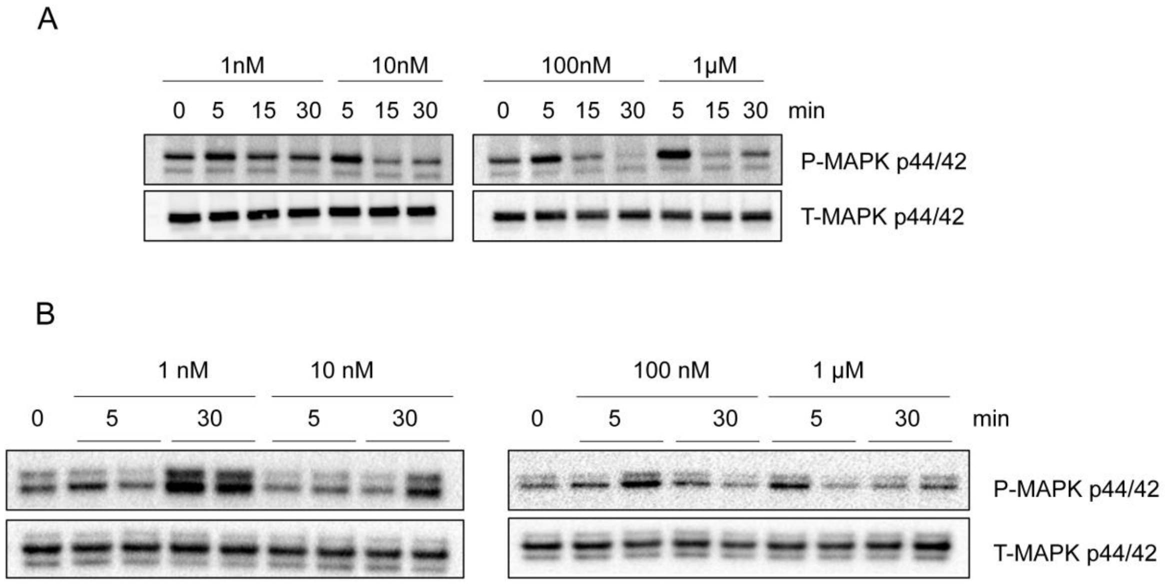

Supplemental Figure 3: HNG increases MAPK p44/42 (ERK 1/2) phosphorylation from 1nM to 100μM. Western blot analysis for phospho- and total-ERK 1/2 in (A) HEK293 and (B) SH-SY5Y cells. Cells were treated with 1nM, 10nM, 100nM, and 1μM HNG for the indicated time periods.

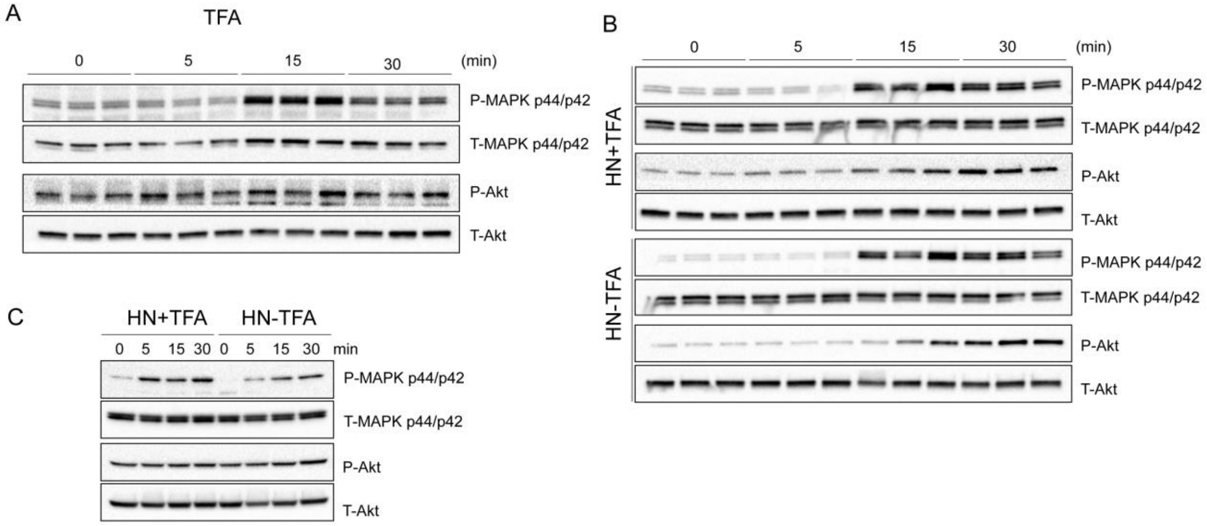

Supplemental Figure 4: Trifluoroacetic acid (TFA) increases MAPK p44/42 (ERK 1/2) phosphorylation. (A) Western blot analysis for phospho- and total-AKT and ERK in SH-SY5Y cells. Cells were treated with 5µM TFA for the indicated time periods. Western blot analysis for phospho- and total-AKT and ERK in (B) SH-SY5Y and (C) HEK293 cells. Cells were treated with 100 µM Humanin with TFA (HN+TFA) and 100 µM Humanin without TFA (HN-TFA) for the indicated time periods.

A

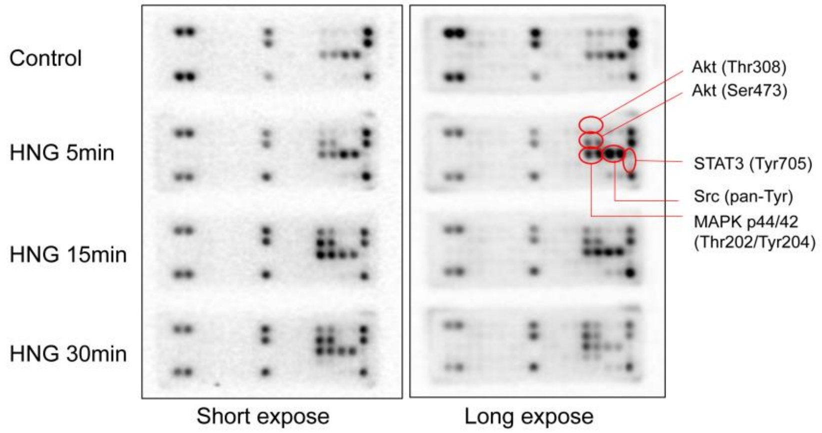

B

Receptor Tyrosine Kinases
EGFR/ErbB1
HER2/ErbB2
HER3/ErbB3
FGFR1
FGFR3
FGFR4
InsR
IGF-IR
TrkA/NTRK1
TrkB/NTRK2
Met/HGFR
Ron/MST1R
Ret
ALK
PDGFR
c-Kit/SCFR
FLT3/Flk2
M-CSFR/CSF-1R
EphA1
EphA2
EphA3
EphB1
EphB3
EphB4
Tyro3/Dtk
Axl
Tie2/TEK
VEGFR2/KDR

Supplemental Figure 5: No evidence for humanin signaling mediated by RTK. (A) SH-SY5Y cells were treated with 100μM HNG for the indicated time periods. The PathScan RTK Signaling Antibody Array images were captured using Bio-Rad ChemiDoc XRS$^+$ imager, with 20 second (short) and 90 second (long) exposure times. (B) A list of RTKs in the antibody array.

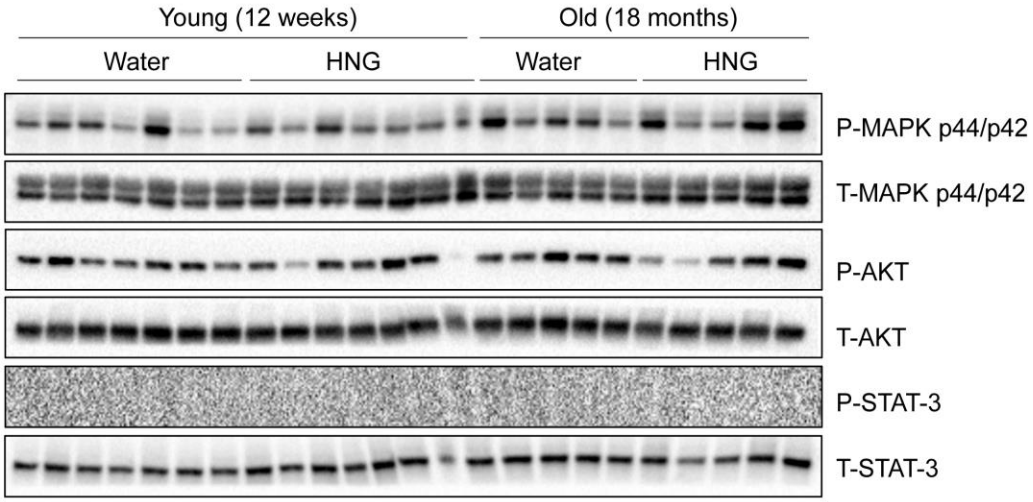

Supplemental Figure 6: No evidence for humanin-mediated AKT, ERK or STAT3 signaling in the hypothalamus. Western blot analysis for phospho- and total-AKT, ERK, and STAT3 in hypothalamus of 12-week-old mice and 18-month-old mice.

A

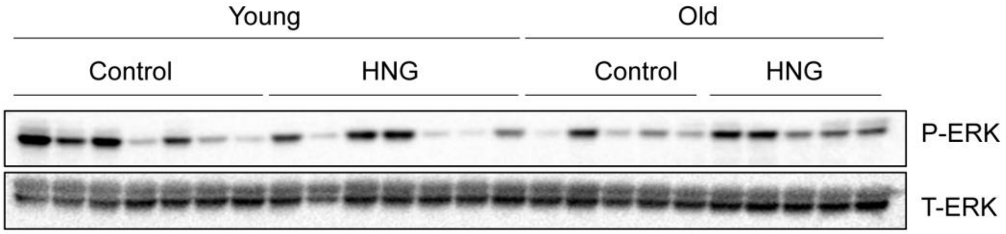

B

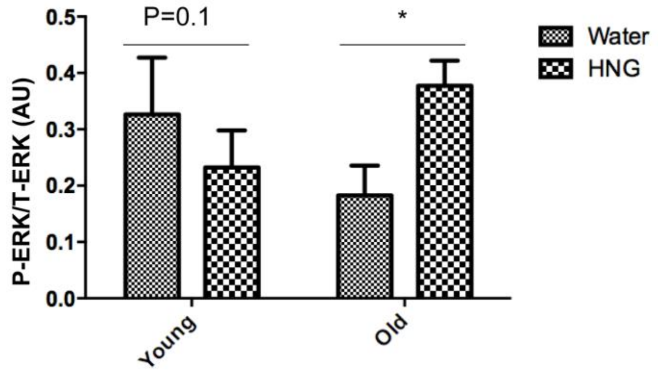

Supplemental Figure 7: HNG increases ERK phosphorylation in the hippocampus of old mice. (A) Western blot analysis for phospho- and total-ERK in hippocampus of 12-week-old mice and 18-month-old mice in the same gel. (B) Quantitation of normalized ERK activation in response to HNG. Total protein was used to normalize protein loading. Data are reported as mean ± SEM (n= 7 for 12-week-old mice, n=5 for 18-month-old mice). *$p<0.05$, ns, not significant.
